# Phenotypic and genotypic characterization of *Candida* species from the oral cavity of healthy individuals in Lublin province, Poland

**DOI:** 10.1080/20002297.2024.2437335

**Published:** 2024-12-09

**Authors:** Patrycja Andrzejuk, Małgorzata Tokarska-Rodak, Andżelika Dyrda, Marta Zarębska

**Affiliations:** a Innovation Research Centre, John Paul II University in Biała Podlaska, Biala Podlaska, Poland; b Faculty of Health Sciences, John Paul II University in Biała Podlaska, Biala Podlaska, Poland

**Keywords:** Genotyping, phenotypic methods, *Candida albicans*, fungi, 25S rDNA, ITS region

## Abstract

**Background:**

*Candida* spp., particularly *C. albicans*, are commonly isolated fungi in the oral cavity. However, their prevalence in healthy participants and their genotype–phenotype relation remains elusive.

**Aim:**

This study aimed to update the information on *Candida* species colonizing the oral cavity of healthy population, identify the most common species, and characterize the intraspecific diversity to determine the genotype-phenotype relationship.

**Methods:**

Oral swabs of healthy participants who declared an absence of oral infection were analyzed. Microbiological methods: chromogenic media, sugar assimilation tests, drug susceptibility, filamentation tests, temperature tolerance analysis, and assessment of biofilm formation ability. Genotyping methods: PCR amplification of the internal transcribed spacer (ITS) region with MspI restriction enzyme digestion and 25S rDNA region.

**Results:**

Of the 500 individuals tested, 130 harbored *C. albicans* in 77%, *C. dubliniensis* in 12%, *Pichia kudriavzevii* (previously *C. krusei*) in 8%, and *Nakaseomyces glabrata* (previously *C. glabrata*) in 3%. The microbiological tests yielded conflicting results. Analysis of the 25S rDNA transposable intron region contributed to the identification of individual *Candida* spp. and intraspecific identification of *C. albicans* genotypes. Genotype A accounted for 70% (*n* = 100) of C. albicans isolates, whereas genotypes B, C, and D (*C. dubliniensis*) accounted for 17%, 9%, and 4% of the isolates, respectively.

**Conclusion:**

The results indicate a complex genotype–phenotype relationship in *Candida* spp. and recommends combining microbiological and molecular methods for the efficient typing of *Candida* spp.

## Introduction

The human microbiome comprises fungal species in addition to bacteria and includes the common genus *Candida*. They have been identified in various host habitats such as the oral cavity, gastrointestinal tract, urogenital tract, and skin surface [1].

*Candida* spp., a genus of fungi present in the oral cavity under physiological conditions, are estimated to account for 45%−65% of the oral microbiome in healthy children and 30%–50% in healthy adults [2]. This genus is a core component of the microbiome, as it can cause oral candidiasis in the event of physiological imbalance (dysbiosis) and, consequently, may lead to systemic infections, especially in patients with immunodeficiencies.

Also the mechanism of the effect of the use of nicotine products on oral *Candida* is debatable. Substances contained in cigarette smoke may affect host immune responses, which may predispose smokers to oral infections [3]. In the literature, some studies revealed that the rate of oral Candidal carriage was higher among smokers compared with non-smokers [4]. No clear differences were noted between female and male smokers.

*Candida albicans*, that causes local and generalized infections, is the most frequently isolated fungal species from the oral cavity. Its ability to occupy several commensal niches is related to its virulence factors. These factors include the potential for morphological transition between yeast and hyphal forms, strong adhesion to host cells, and biofilm formation ability [5]. As organized structures, biofilms consist of closely adherent cells of one or several different species of microorganisms that are bound to the substrate and embedded in the extracellular matrix. In human body, 80% of biofilm formation is presumably associated with fungal or bacterial infections, given the durability of these structures [1,5].

Surface colonization by *Candida* when accompanied by biofilm formation is a multistage process. The first stage involves the adhesion of the cells to the substrate, providing structural stability. This process depends on many factors, including physicochemical interactions between microbial cells and the surface, reactions between specific fungal proteins and polysaccharides, environmental factors, chemical composition of cell membranes, specific genes, and substrate roughness [6]. The second stage is proliferation, which involves the formation of filaments, the use of which elongates the yeast cells, forming filamentous hyphae. *C. albicans* fungi can form hyphae both in planktonic cultures and at the biofilm formation stage. During this process, the fungal cells alter their morphology and become highly stable within the host cells. Hyphal growth is also regulated by transcriptional regulators and structural proteins, and the deletion of any of these elements reduces or eliminates growth [7]. The next stage is the biofilm maturation phase, in which the production of hyphae is accompanied by the secretion of extracellular polymeric substances [8,9]. The extracellular matrix, mainly composed of glycoproteins (50%), acts as a physical protective barrier to ensure the structural integrity of the biofilm. Without a matrix, fungal cells become desiccated and lose their nanomechanical properties [10]. Yeast-like cells are released from mature biofilms, leading to the formation of new structures in distant locations, leading to infection dissemination [11].

Biofilms formed by various *Candida* species differ in the complexity of their extracellular matrix structures and compositions. Mature *C. albicans* biofilms are distinguished from those produced by other *Candida* spp. because of their extensive hyphal networks. Both *C. albicans* and *C. dubliniensis*, which are difficult to distinguish using a single biochemical test, produce blastospores, hyphae, and pseudo-hyphae. Blastospores and pseudohyphae are also formed by *C. parapsilosis*, *Pichia kudriavzevii* (previously known as *C. krusei)*, and *C. tropicalis*. The fungus *Nakaseomyces glabrata* (previous *C. glabrata)* produces only blastospores [7,12,13].

*C. albicans* isolates are problematic as they were inconsistent in their ability to form biofilms when tested using patient samples. There are five *C. albicans* genotypes, A, B, C, D, and E, and the identification of these isolates is also troublesome. Genotype A is considered predominant, and genotype D is considered to be associated with *C. dubliniensis* [14]. Determining the genotype–phenotype relationship could predict whether a specific genotype is more likely to cause an infection. Therefore, the present study focused on determining the relationship between the *C. albicans* genotype and its phenotypic traits and biofilm-forming ability. We aimed to update our knowledge on *Candida* species colonizing the oral cavity of a healthy population. We isolated the most commonly present species and characterized the intraspecific diversity to determine the genotype–phenotype relationship.

## Materials and methods

### Sample collection

The study group consisted of 500 participants (288 females, 212 males) aged 18–90 years (average age, 39 years; σ = 16.79). Oral swabs were obtained from healthy volunteers with no symptoms of infection or coexisting diseases from a local community. The samples were collected between June 2021 and September 2022 in Lublin province. A questionnaire was administered to the participants to collect information on current and past oral infections, treatment methods, use of nicotine products (smoking cigarettes, e-cigarettes, and nicotine gums), morbidities, and metric data (Table 1). The study design was approved by the Bioethics Committee at Pope John Paul II State Higher School (currently John Paul II University in Biała Podlaska) (consent No. 3/2021), and all participants provided informed consent. The sample was collected using sterile swabs with a transport medium by rubbing the insides of the cheek for 15 s on each side. The sample was cultured onto Sabouraud dextrose (Argenta, Poznań, Poland) media for 24 h at 35°C. The colonies were plated on chromogenic media for further molecular analyses. Samples were banked using microbanks (Mast Group, Reinfeld, Germany), a ready to use system designed for the storage of bacterial and fungal cultures and stored at − 20°C until recovery.

**Table 1.** Participant characteristics.

	Females	Males
Number of participants	288	212
Aged 18–30 years	42% (121/288)	34% (72/212)
Aged 30–60 years	48% (138/288)	48% (102/212)
Aged above 60 years	10% (29/288)	18% (38/212)
Use of nicotine products	49% (141/288)	55% (117/212)
Country of origin	Poland	Poland
Current oral infections	0%	0%
Existing diseases	0%	0%

## Phenotypic methods

### Chromogenic agar culture

After the fungi grew on Sabouraud medium, they were cultured onto chromogenic media (CHROMagar, GRASO, Poland) and incubated at 35°C for 48 h. This medium allows for the qualitative identification of yeast-like fungi of the genus *Candida*. On CHROMagar, *C. albicans* appears green, whereas *C. tropicalis* and *C. krusei* (currently *P. kudriavzevii*) appear metallic blue and pink, respectively. The color of *C. glabrata* (currently *N. glabrata*) ranges from violet to pink to brown, and other species are seen in shades of white to mauve-pink. Among the fungi that grew, only *C. albicans* was selected for further analyses using other phenotypic methods. Because the final results of the individual phenotypic tests were subject to unclear interpretations, a variety of analyses were performed. All results were reviewed by two independent researchers.

### Sugar assimilation test

To validate the identified species in all samples, we used the AUXACOLOR 2 (Bio-Rad, Warsaw, Poland) test, a colorimetric test based on sugar assimilation. In this test, sugar utilization for yeast growth is visualized by a color change in the pH indicator from blue to yellow. Test inoculum (McFarland standard of 1.5, 100 μL), prepared from a 24-h culture, was applied to all the wells in the test apparatus and incubated at 30°C. Readings were taken after 24 and 48 h and after 72 h in case of doubt. Test results were interpreted according to the manufacturer’s recommendations.

### Temperature tolerance test

For the temperature tolerance test, the cultures were incubated on Sabouraud solid media at 42°C, and observations were made after 24, 48, and 72 h. The test is useful to distinguish between *C. albicans* and C. *dubliniensis*. While *C. albicans* can grow at 42°C, owing to their ability to survive unfavorable conditions, *C. dubliniensis* cannot.

### Filamentation test

Fungal cells, adjusted to a McFarland standard of 2 by adding 0.5 mL human serum, were incubated at 37°C for approximately 2.5 h. Subsequently, the material was centrifuged for 3 min at 1006 × g, the supernatant was removed, and the samples were observed under a microscope (Nikon Eclipse 80i, Warsaw, Poland). The presence of filaments, that is structures characterized by non-septate cell expansion (as opposed to pseudohyphae) and lack of constriction from the mother cell, confirms the identification of *C. albicans* species (Gnat 2022).

## Genotypic methods

### Genomic DNA extraction

DNA isolation was performed using a Genomic Mini Kit (A&A Biotechnology, Gdańsk, Poland) according to the manufacturer’s instructions. After 24 h of incubation, an inoculum with approximately 1.5 McFarland standard was prepared from Sabouraud’s medium culture, transferred to Eppendorf tubes, and placed in a thermoblock heated to 70°C for 12 h to facilitate cell lysis.

### Restriction fragment length polymorphism analysis

Molecular identification of the strains began with the identification of the internal transcribed spacer (ITS) region. The ITS1–5.8S-ITS2 region is characterized by relatively conserved sequences, which facilitate correct sequence alignment, and high variability, thereby ensuring the utility of non-homologous sequences as restriction fragment length polymorphism analysis markers. The following primers were selected for amplification of the targeted ribosomal DNA: ITS-1 (5′-TCCGTAGGTGAACCTGCGG-3′) and ITS-4 (5′-TCCTCCGCTTATTGATATGC-3′). The PCR was carried out in a total volume of 50 µL, and the reaction mixture contained 2 µL of template DNA, 0.5 µL of each primer (25 µM), 1.25 µL of dNTPs, 5 µL of 10× PCR buffer, and 0.5 µL of Taq DNA polymerase (0.5 U). Initial denaturation was carried out for 5 min at 94°C, followed by denaturation at 94°C for 1 min, annealing at 56°C for 1 min, and chain elongation at 72°C for 1 min in 30 cycles. The final elongation step was performed at 2°C for 7 min. Subsequently, restriction digestion was performed using the MspI restriction enzyme (10 U/µL) (Thermo Scientific, Warsaw, Poland), in a total volume of 25 µL; the digestion was achieved by incubating 21.5 µL of the PCR products with 2.5 µL of the buffer and 1 µL of the MspI enzyme. Restriction fragments were separated using 2% agarose gel electrophoresis in TBE buffer (EURx, Gdańsk, Poland) at 100 V for 1 h and stained with Gel Stain (Syngen, Wrocław, Poland) for visualization.

### Genotyping using 25S rDNA

Tests for molecular differentiation of various *C. albicans* strains and between *C. albicans* and *C. dubliniensis* were carried out based on the 25S rDNA sequence. The sizes and lengths of the 25S rDNA intronic regions were determined to identify specific genotypes (A–E) of *C. albicans*. The primers CA-INT-L (5′-ATAAGGGAAGTCGGCAAAATACCGTAA-3′) and CA-INT-R (5′- CCTTGGCTGTGGTTTCGCTAGATAGTAGAT-3′) were used for the sequence detection. The amplification reaction was carried out using 12.5 µL of Hot Start MIX (A&A Biotechnology, Gdańsk, Poland), 2.5 µL of each primer (20 pmol), and 5 µL of genomic DNA in a total volume of 25 µL. The following conditions were used for PCR: initial denaturation at 94°C for 3 min, 30 cycles of denaturation at 94°C for 30 s, annealing at 60°C for 15 s, extension at 72°C for 1 min, and final elongation step at 72°C for 10 min (Mashaly and Zeid, 2022). The PCR products with expected sizes (genotype A, 450 bp; genotype B, 840 bp; genotype C, 450 bp and 840 bp; genotype D, 1040 bp; genotype E, 1400 bp) were visualized using electrophoresis on a 1.5% agarose gel, using TBE buffer and Gel Stain, at 100 V for 1 h.

Data were analyzed using Statistica 13 program (Biala Podlaska, Poland). Mean, frequency, and standard deviation values, Chi-square statistic, critical value, p-value were determined. *p* < 0.05 was considered statistically significant.

## Downstream analyses

### Drug susceptibility assessment

Antifungal susceptibility testing was performed on isolates confirmed as *C. albicans* (genotypes A, B, or C) to the commonly used antifungals anidulafungin, amphotericin B, micafungin, posaconazole, voriconazole, itraconazole, and fluconazole. To examine the drug susceptibility profile, minimum inhibitory concentrations (MICs) of the selected agents were determined using a microbroth dilution test in a dried 96-well microplate format. Each well contained antifungal agents at appropriate dilutions and a calorimetric indicator (Sensititre YeastOne; Thermo Scientific, Warsaw, Poland). The plate was incubated at 35°C for 24 or 48 h in case of doubt. The lowest concentration of the antifungal agent that inhibited fungal growth (as evidenced by the lack of color change) was recorded. Strains were considered sensitive or resistant according to EUCAST recommendations [15].

### Biofilm formation analysis

Cultures incubated at 35°C for 48 h were used for biofilm observation. A microscope slide coated with gelatin was placed in a sterile container and supplemented with 2 mL of the fungal inoculum (with a McFarland standard of 4) and 2 mL of Sabouraud dextrose liquid medium (Oxoid, Pol-Aura, Warsaw, Poland). After incubation, non-adherent and planktonic cells were removed by washing with PBS (phosphate-buffered saline) (1.5 mL). Biofilms were visualized and analyzed using a Nikon optical microscope (Eclipse 80i model) equipped with a Nikon DS-Fi1c camera at 40× magnification. Most biofilms formed under *in vitro* laboratory conditions have only a few layers and cells that adhere well to the substrate and are resistant to repeated washing. The degree of biofilm formation by each isolate was determined as follows. After rinsing, a biofilm occupying more than 80% of the field of view with complex structures was defined as a strong biofilm; if it occupied 50% of the area with few cells, it was described as an intermediate biofilm, and a biofilm with an occupancy below this value was identified as a weak biofilm.

## Results

*Candida* spp. were detected in the oral swab samples of 130 of the 500 participants, indicating that 26% of individuals harbored the fungi (Table 2). Of the 130 *Candida-*positive samples, 77% (100/130) carried *C. albicans*, 12% (16/130) contained *C. dubliniensis*, 8% (10/130) contained *P. kudriavzevii*, and 3% (4/130) contained *N. glabrata*, as revealed by phenotypic analyses such as the CHROMagar identification. In this study, no more than one species was isolated from a single individual.

**Table 2.** Characteristics of the 130 individuals who harbored *Candida* spp.

	Females	Males	Statistical calculations
Samples harboring Candida spp.	63% (82/130)	37% (48/130)	
Aged 18–30 years	38% (31/82)	32% (15/48)	
Aged 30–60 years	54% (44/82)	49% (24/48)	
Aged above 60 years	8% (7/82)	19% (9/48)	
Use of nicotine products	17% (14/82)	32% (15/48)	χ^2^ = 3.519 *df* = 1 α = 0.05 p-value = 0.061

Statistical analyses were performed used Statistica 13 to determine significant differences between male and female participants who tested positive for *Candida* spp. and used nicotine. The null hypothesis assumed no significant difference in the distribution of the number of individuals harboring *Candida* spp. and nicotine use between men and women. The values of the chi-square statistic (χ^2^ = 3.519) were compared with the critical value (3.841) at *df* = 1; at the pre-determined significance level (α = 0.05), there was no difference (*p* = 0.061) in the prevalence of *Candida* spp. and nicotine use between men and women.

Further phenotypic identification tests were performed on the 100 isolates identified as *C. albicans* using chromogenic media. Results are summarized in Table 3.

**Table 3.** Phenotypic test results using the AUXACOLOR sugar assimilation, temperature tolerance, filamentation, and sensititre YeastOne tests for the 100 isolates initially classified as *C. albicans.*

	C. albicans	C. dubliniensis
AUXACOLOR sugar assimilation test	97% (97/100)	3% (3/100)
Temperature tolerance test	93% (93/100)	7% (7/100)*
Filamentation test	97% (97/100)	3% (3/100)**
Sensititre YeastOne test	100% (100/100)	0% (0/100)

*Results of three AUXACOLOR tests considered; **For one isolate, the results were validated using temperature tolerance and AUXACOLOR tests as well.

**Figure 1.**
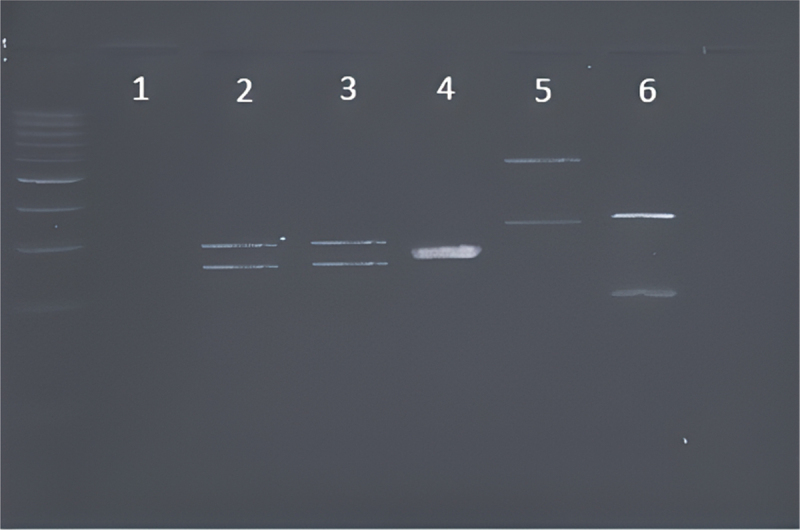
Band patterns of the PCR products of the ITS1–5.8S-IT2 region after MspI restriction digestion for *Candida* spp. visualized using gel electrophoresis; lane 1, negative control; lane 2, *C. albicans;* lane 3, *C. dubliniensis;* lane 4, *P. kudriavzevii;* lane 5, *N. glabrata;* and lane 6, *C. tropicalis.*

PCR products, of sizes ranging from approximately 510 to 870 bp, specific for *Candida* spp. were identified by analyzing the ITS region. After cleaving the ITS region with the MspI restriction enzyme, two polymorphic bands were obtained for *C. albicans*, *C. dubliniensis*, *N. glabrata*, and *C. tropicalis*, and one band was obtained for *P. kudriavzevii*. The similarity in the restriction patterns of *C. albicans* and *C. dubliniensis* prevented proper differentiation of the two strains (Figure 1).

**Figure 2.**
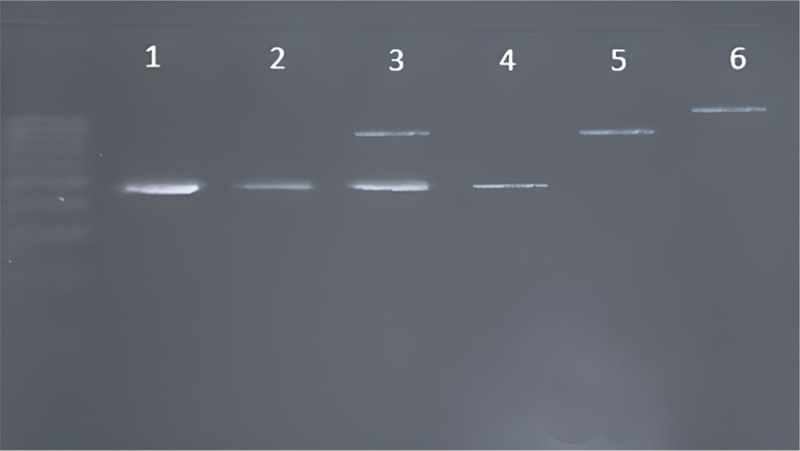
Genotyping DNA band pattern profiles. Lanes 1, 2, and 4, *C. albicans* genotype A; lane 3, *C. albicans* genotype C; lane 5, *C. albicans* genotype B; lane 6, *C. dubliniensis/C. albicans* genotype D.

All 100 strains that were isolated and tentatively identified as *C. albicans* and/or *C. dubliniensis* were subjected to PCR analysis based on the 25S rDNA sequence for genotyping; a single product was obtained for genotypes A (450 bp) and B (840 bp), and a double product was obtained for genotype C (450 bp and 840 bp). The following genotypes were identified (Figure 2): genotype A (70%), B (17%), C (9%), and D (*C. dubliniensis*, 4%). All analyses for genotype D yielded a band identical to that of *C. dubliniensis* (1080 bp), which contradicted the results obtained using sugar assimilation and filamentation tests. Genotype E was not detected in the present study. Further, the results revealed no relationship between the occurrence of a given genotype and nicotine use.

The 96 isolates confirmed as *C. albicans* (genotype A, B, or C) were tested for antifungal susceptibility. The results are summarized in Table 4, along with the MICs of the agents used for testing. All samples identified as genotypes B or C were susceptible to all the antifungal drugs. Genotype A samples were highly resistant, with 57% (40 of 70) of the isolates showing resistance to anidulafungin. In addition, resistance to posaconazole, itraconazole, and voriconazole was observed in 5 (7.1%), 15 (21.4%), and 4 (5.71%) isolates, respectively. However, all the genotype A samples were susceptible to fluconazole, micafungin, and amphotericin B.

**Table 4.** MIC values (mg/l) of the antifungal agents against the tested *Candida albicans* strains (genotype A, B, or C).

	Candida albicans species			
	*Genotype A*	*Genotype B*	*Genotype C*	
Number of isolates (*n* = 96)	70 (73%)	17 (18%)	9 (9%)	MIC breakpoint (mg/L) S ≤ 2 R > 4
Antifungal agents used for testing		MIC values (mg/L)		
FLUCONAZOLE min–max SD x¯ CV	0.12–1 0.20 0.27 0.76	0.12–0.5 0.10 0.29 0.34	0.12–1 0.25 0.37 0.67	
POSACONAZOLE min–max SD x¯ CV	0.15–0.8 0.02 0.02 0.94	0.015–0.3 0.01 0.02 0.65	0.008–0.3 0 0.02 0.28	S ≤ 0.06 R > 0.06
ITRACONAZOLE min–max SD CV	0.015–0.5 0.02 0.04 0.44	0.015–0.06 0.01 0.03 0.38	0.015–0.06 0.02 0.04 0.42	S ≤ 0.06 R > 0.06
MICAFUNGIN min–max SDx¯ CV	0.008–0.03 0.01 0.01 0.51	0.008–0.015 0 0.01 0.29	0.008–0.015 0 0.01 0.25	S ≤ 0.016 R > 0.016
VORICONAZOLE min–max SDx¯ CV	0.008–0.12 0.11 0.04 2.95	0.008 0 0.01 0	0.008–0.015 0 0.01 0.25	S ≤ 0.06 R > 0.25
ANIDULAFUNGIN min–max SDx¯ CV	0.015–0.06 0.02 0.04 0.52	0.015–0.03 0.01 0.02 0.29	0.015–0.03 0,01 0,03 0,41	S ≤ 0.03 R > 0.03
AMPHOTERICIN B min–max SDx¯ CV	0.25–1 0.24 0.45 0.54	0.25–0.5 0.10 0.29 0.36	0.25–1 0.23 0.44 0.52	S ≤ 1 R > 1

SD: standard deviation (mg/L); x¯: arithmetic average (mg/L); CV: coefficient of variation (%); S (susceptibility breakpoint); R (resistance breakpoint).

**Figure 3.**
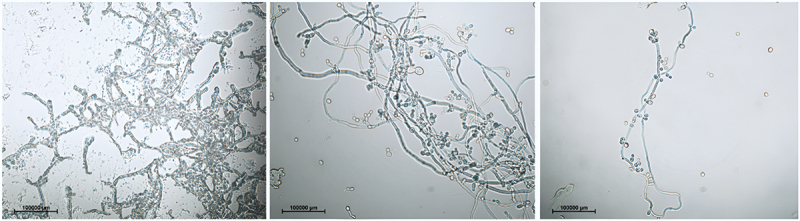
Images of biofilms formed *in vitro* by *C. albicans* genotype a isolates. (A) Strong biofilm, (B) intermediate biofilm, (C) weak biofilm.

Among the isolates classified as *C. albicans*, 65% (62 of 96) formed biofilms (Figure 3) and 35% (34 of 96) were present in the planktonic form. The genotype distribution of the isolates in the biofilm-forming group was as follows: 82% (51 of 62) of genotype A, 11% (7 of 62) of genotype B, and 7% (4 of 62) of genotype C.

Six strains, with five representing genotype A (83%) and one representing genotype C (17%), formed strong biofilms. Genotype D isolates did not form biofilms.

## Discussion

The development and recurrence of fungal infections of the oral cavity are closely associated with the presence of *Candida* spp. in the microbiome. The present study revealed *Candida* yeast colonization in the oral cavity of 26% of the examined participants, which is approximately 13% lower than the prevalence reported by Gerós-Mesquita et al. [16] in a similar study with healthy participants. In the study by Szymańska et al. [17], the incidence of yeast in the oral cavity of healthy participants was 30%.

*C. albicans* is the most common species in this environment owing to its features such as high pathogenicity, virulence, and adaptability [2,18,19]. Our findings re-affirmed this result, with *C. albicans* still being the most frequently (77%) isolated species of this genus. The high prevalence in healthy participants who declared no oral infections indicates that its presence in patients with compromised immunity or chronic inflammation in the oral cavity may lead to the development of infection and colonization of other niches in the body.

Our research hypothesis assumed a significant influence of external factors on colonization in the oral cavity but in research we did not observe a correlation between increased yeast colonization of the oral cavity and the use of nicotine products. Among people without *C. albicans* colonization, the majority of participants smoked across all the age groups. In addition typically, the functions of the innate immune system are impaired in elderly individuals aged >60 years. Surprisingly, in our study, *C. albicans* isolates were mostly identified in samples collected from participants aged 30–60 years, with a slight predominance in females.

Proper identification of the pathogen is important for the implementation of appropriate treatment and antibiotic therapy. Different methods have been described for *Candida* spp. identification and genotyping [14,20,21]. The choice of typing method depends on the nature and aims of the molecular study, and the efficiency of the technique is determined by its discrimination power, repeatability, accuracy, ease of performance, and interpretability of results. In the present study, phenotypic methods, even when using multiple techniques, may yield ambiguous results and cannot be performed selectively. We demonstrated that a single method is insufficient for the identification of *Candida* fungi, especially to discriminate between *C. albicans* and *C. dubliniensis*, owing to their common phenotypic traits. However, microbiological methods have limitations. Often, the interpretation of results is visual (based on color readings), and the results may be subjective. These disadvantages of phenotype-based techniques for *Candida* spp. identification were confirmed in the present study. Results must be reproducible and interpreted identically, regardless of the handling team, and hence, molecular methods, such as PCR-based methods, are currently used to complement microbiological methods and provide accurate results. In our study, the species identified as *C. albicans* using microbiological techniques was re-assigned as *C. dubliniensis* on genomic analysis.

Progress in molecular biology has facilitated its use in pathogen typing. The distinction of *Candida* spp. in the oral cavity based on the ITS region and digestion of PCR products with the MspI restriction enzyme revealed patterns for the easy identification of *P. kudriavzevii*, *N. glabrata*, and *C. tropicalis*. The electrophoretic results for *C. albicans* and *C. dubliniensis* were almost identical. This is not surprising considering the limitations associated with phenotypic identification. These results suggest that the strains are strongly interrelated, as confirmed by other researchers such as Zahir and Himratul-Aznita [22]. In the present study, ITS method proved efficient for the analysis of strains in the microbiome of healthy participants, as it revealed the interspecific differentiation of *P. kudriavzevii*, *C. tropicalis*, *N. glabrata*, and *C. albicans/C. dubliniensis*. However, the drawback with respect to the identification of *C. albicans* and *C. dubliniensis* could have been overcome by the selection of a different restriction enzyme, for example BlnI, as shown by Shokohi et al. [23].

Genotypic methods also facilitate the identification of the same or closely related strains in independent isolates, assessment of their microevolution and variability, and recognition of unrelated strains. A relatively easy, quick, and reliable genotyping method was used to analyze changes in the length of the 25S rDNA transposable intron, which allowed interspecific and intraspecific differentiation of *C. albicans* based on band patterns. The high variability and intraspecific diversity of *C. albicans* was evidenced by the presence of genotypes A, B, and C. The findings from the present study showed that the oral cavity of most healthy participants were colonized by genotype A (70%), followed by genotypes B (17%), C (9%), and occasionally genotype D identified as *C. dubliniensis* (4%). Further, no two genotypes were simultaneously identified in any participant. Similar results have been reported by Mashaly and Zeid [24] and She et al. [25]. Compared with the results of our research, other results were obtained by Tantivitayakul et al. [14]. In this report found that genotype B was the most common genotype in a pool of participants from Thailand. Researchers suggest the influence of geographical location on the distribution of genotypes.

*C. albicans* isolates classified as genotypes B and C were susceptible to all the antifungal agents used for testing, including all the echinocandins, whereas genotype A was resistant to anidulafungin, posaconazole, itraconazole, and voriconazole. This may be related to the prevalence of genotype A in the healthy population and its higher adaptability than those of the less common genotypes B and C, leading to acquired resistance. Resistance of the common *C. albicans* genotype A to azole and echinocandins antifungals may contribute to treatment failure [26]. This is due to the fact that they constitute the basic groups of drugs used in potential use in therapy of Invasive fungal disease. Further, our findings differ from those of Kumar et al. [27], in which *C. albicans* genotype B was resistant to most echinocandins. A low percentage of *C. albicans* is reportedly resistant to antifungal drugs by Kessler et al. [28]. The highest resistance rates were observed against miconazole and econazole, regardless of the presence of systemic disease. Comparing this with the results of our study, the differences may be caused by the geographical region of the carriers (Brazil/Poland) or the genotypes of the individual strains. This may also be attributed to the diversity of microorganisms colonizing the oral cavity.

rDNA analyses, in combination with the assessment of other pathogenic traits such as filamentation, colony morphology, and drug sensitivity, revealed that high intraspecific diversity was related to the ability to form biofilms. As confirmed in the present study, each genotype was characterized by different biofilm-forming abilities. Biofilms were formed by 43% (highest proportion) of genotype A, 11% of genotype B, and 7% of genotype C isolates, with strong substrate adhesion exhibited by biofilms formed by the first and third genotypes. Additionally, genotype A exhibited the highest filamentation ability among all genotypes, which could be associated with the extreme ease of creating complex structures by these microorganisms. Nevertheless, isolates that could not form biofilms were also observed, even those belonging to genotype A, which might be related to the individual characteristics acquired from the host.

## Conclusions

The present study showed that microbiological methods have limitations and that they should be combined with molecular methods for efficient typing of *Candida* spp. ABC genotyping revealed that the predominating genotype is A among the *C. albicans* strains isolated in our study, which is probably associated with its other traits: extensive colonization of the oral cavity of healthy people, ease of biofilm formation, strong adhesion to the substrate, high resistance to antifungal drugs, intense filamentation, and high adaptability. The importance of the genotype-phenotype relationship in *Candida albicans* genotype A is well known complemented by studies that have provided deeper insight into the pathogenicity and development of fungal infections with the strains most commonly found in the oral cavity, even in healthy individuals.
